# Modelling and Experiment Based on a Navigation System for a Cranio-Maxillofacial Surgical Robot

**DOI:** 10.1155/2018/4670852

**Published:** 2018-01-10

**Authors:** Xingguang Duan, Liang Gao, Yonggui Wang, Jianxi Li, Haoyuan Li, Yanjun Guo

**Affiliations:** ^1^Intelligent Robotics Institute, Beijing Institute of Technology, Beijing 100081, China; ^2^Beijing Advanced Innovation Center for Intelligent Robots and Systems, Beijing 100081, China; ^3^Beijing Institute of Technology, Beijing 100081, China

## Abstract

In view of the characteristics of high risk and high accuracy in cranio-maxillofacial surgery, we present a novel surgical robot system that can be used in a variety of surgeries. The surgical robot system can assist surgeons in completing biopsy of skull base lesions, radiofrequency thermocoagulation of the trigeminal ganglion, and radioactive particle implantation of skull base malignant tumors. This paper focuses on modelling and experimental analyses of the robot system based on navigation technology. Firstly, the transformation relationship between the subsystems is realized based on the quaternion and the iterative closest point registration algorithm. The hand-eye coordination model based on optical navigation is established to control the end effector of the robot moving to the target position along the planning path. The closed-loop control method, “kinematics + optics” hybrid motion control method, is presented to improve the positioning accuracy of the system. Secondly, the accuracy of the system model was tested by model experiments. And the feasibility of the closed-loop control method was verified by comparing the positioning accuracy before and after the application of the method. Finally, the skull model experiments were performed to evaluate the function of the surgical robot system. The results validate its feasibility and are consistent with the preoperative surgical planning.

## 1. Introduction

Cranio-maxillofacial region is the most exposed part of the human body and plays an important role in the maintenance of facial feature, speaking, swallowing, chewing, facial expressions, and so on. The congenital malformation, traumatic defect, and trauma after tumor resection of the cranio-maxillofacial region are the common clinical diseases. These diseases will bring great psychological pressure and inconvenience to people's life and work. In order to reduce the trauma of cranio-maxillofacial surgery for patients, the minimally invasive treatment is often adopted. And the cranio-maxillofacial puncture manually by surgeons is typical of minimally invasive treatment [1].

With the fast development of science and technology, computer-assisted surgery becomes the necessary supportive tool for diagnosis, operation planning, and treatment in medicine services [2–5]. In clinical routine, surgeons have been already supported by various computer-assisted devices such as the 3D reconstruction system, preoperative planning system, and intraoperative navigation system, especially the navigation system, which has brought great convenience for surgery and attracted increasing attentions. The navigation system is composed of stereotactic medical imaging technology, computer technology, artificial intelligence technology, and minimal invasive surgery [6]. In 1881, Zernov developed a brain-measuring instrument, which is the earliest surgery navigation records. In 1908, Horsley and Clarke created brain stereotactic technology [7]. In 1990, the United States launched the StealthStation, which is the first commercial surgery navigation system in the world and can be used in the human body. After that, the Stryker (Stryker-Leibinger, Kalamazoo, MI, USA), VectorVision (BrainLab, Munich, Germany), StealthStation (Medtronic-Xomed, Jacksonville, FL, USA), and other products have been launched, and these products can be competent in many navigation surgeries [8]. At present, the navigation system commonly used in surgery is mainly divided into the following categories, that is, the optical navigation system, ultrasonic navigation system, and electromagnetic navigation system. These navigation systems have different advantages, and the optical navigation system becomes the mainstream for its ease of use, simple operation, and high accuracy in the surgical navigation system. The navigation system establishes the dynamic relationship between virtual digital images and actual operation structure and solves the problems of clinical surgeons that need to be solved urgently, including preoperative virtual surgery planning and intraoperative lesion accurate positioning. The computer-assisted technology based on the navigation system has improved the flexibility, accuracy, and prognosis of surgery largely [9, 10].

Furthermore, the robot technology has been widely used in cranio-maxillofacial surgery for its flexibility of control, stability, and high accuracy [11, 12]. In 1994, Kavanagh carried out preclinical tests in the field of oral and maxillofacial surgery using the image-guided robot system on the temporal bone for the first time [13]. In 1998, Lueth et al. developed the first interactive surgical robot (OTTO) for cranio-maxillofacial surgery. The robot was used for inserting nonflexible catheters and implanting bone fixtures [14]. In 2006, Bale et al. installed a frameless stereotactic device on a six-degrees-of-freedom mechanical arm for the ablative treatment of trigeminal neuralgia. The average positioning accuracy can reach 1.31 ± 0.67 mm in the model, body, and clinical trials [15]. In other surgeries, many robot systems have been developed, such as Robopsy, B-Rob I, and ZEUS.

To the best of our knowledge, although robot-assisted technology has been applied in cranio-maxillofacial surgery [16], its use for biopsy of skull base lesions and radioactive particle implantation of skull base malignant tumors has not yet been reported. And the function of cranio-maxillofacial puncture robot is single, which is designed for a certain operation, such as radiofrequency thermocoagulation. This makes it necessary for surgeons to switch and adapt to different surgical systems for different surgeries, which brings inconvenience to the operation. However, according to clinical experience, some cranio-maxillofacial surgery procedures have similarities; that is, puncture is needed to be done. Therefore, in order to make the surgical robot universal, a robot system that can be used in a variety of punctures is needed.

In robot puncturing surgery, the needle can be navigated by computed tomography (CT), ultrasound, or magnetic resonance image (MRI) to ensure the positioning accuracy of the needle. The iSYS1 robot system [17], AcuBot system [18], and PAKY-RAM robot [19] guided needle puncture through CT fluoroscopy to achieve high positioning accuracy. But CT fluoroscopy exposes the patient to radiation and does some harm to the patient's health. Hong et al. proposed an ultrasound-driven needle insertion robot for percutaneous cholecystostomy, which is capable of modifying the needle path by real-time motion compensation through visual servo control before needle insertion [20]. But ultrasound can provide only two-dimensional (2D) anatomical information with poor quality. MRI is nonirradiating, but its application in a robot surgery system is limited because of its cost, tunnel size, and material compatibility constraints [21]. In order to obtain better positioning accuracy, the needle position has to be checked repeatedly. Therefore, an accurate and safe navigation system is required during operation.

Although the structure of the cranio-maxillofacial region is complex and the forms of lesions are different, some experienced surgeons performing the operation could reduce most of the potential risks and have good prognosis by using a commercialized surgery navigation system. For surgeons, flexible and precise puncture can help them reduce the operation time, risk, and fatigue. Then, a new type of a cranio-maxillofacial surgery robot system is developed to assist surgeons in performing a variety of cranio-maxillofacial puncture surgeries, such as biopsy of skull base lesions, radiofrequency thermocoagulation of the trigeminal ganglion, and radioactive particle implantation of skull base malignant tumors. This paper aims to relate the components of the entire robot system and to realize the interaction among the robot, navigation system, and patient. The “kinematics + optics” hybrid motion control method is proposed in this paper, which can form a closed-loop system to improve the positioning accuracy of the system. Then, the robot can move accurately along a predetermined trajectory and assist surgeons to complete the corresponding cranio-maxillofacial surgery.

## 2. System and Model

### 2.1. System Composition

**Figure 1:**
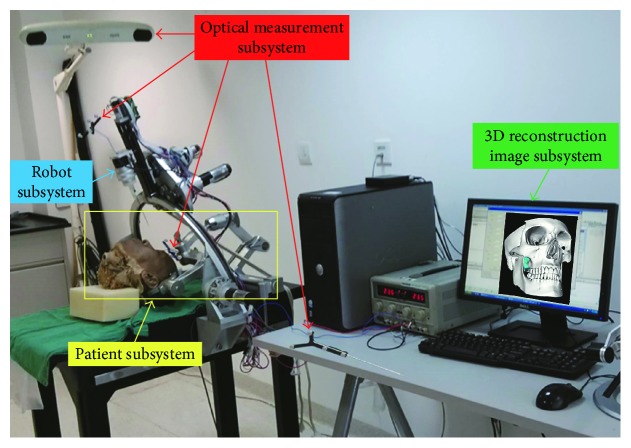
The cranio-maxillofacial surgery robot system.

The cranio-maxillofacial surgery robot system (Figure 1) can be divided into four parts according to the function, including the patient subsystem, robot subsystem, optical measurement subsystem, and 3D reconstruction image subsystem [22]. The patient subsystem is the object of surgical treatment, and the head of the patient is fixed and adjusted through the head clamp to coincide with the operation. The robot subsystem is a frame structure with five degrees of freedom, and the working space can reach 300 × 400 × 400 mm, which can meet the needs of the operation. By switching the end effector, the robot subsystem can perform a variety of operations, such as biopsy, radiofrequency thermocoagulation, and radioactive particle implantation. The optical measurement subsystem mainly realizes the spatial registration between each subsystem and real-time tracking in the operation. The 3D reconstruction image subsystem can obtain a 3D image of the cranio-maxillofacial region by CT scanning and reconstruction, which is applied to the entire operation, and provides guarantee for preoperative operation planning, intraoperative navigation, and postoperative verification.

Here, we mainly focus on the optical measurement subsystem. The optical measurement subsystem mainly consists of a set of optical measuring instruments (NDI Polaris, Canada). The optical measuring instrument includes an optical tracker, two passive rigid bodies, and a passive probe. The two passive rigid bodies are fixed on the robot and patient's head, respectively. The passive rigid bodies and probe can create the corresponding coordinate system by the position of four reflecting balls attached to them. The optical tracker can obtain the position of the reflecting balls by transmitting the infrared signal. In the range of measurement, the accuracy of the optical measuring instrument is 0.25 mm RMS.

In this paper, the relationship between the four subsystems is described, which are used to guide the movement of the robot arm. The spatial registration is firstly to realize the transformation of coordinate spaces between the four subsystems, so as to realize the integration of them. Secondly, the end effector of the robot can be controlled to a certain position in the robot work space through the hand-eye coordination method. In addition, the positioning accuracy of the end effector of the robot can be improved by the “kinematics + optics” hybrid motion control method.

### 2.2. System Model

#### 2.2.1. Navigation Spatial Registration Based on an Improved ICP Algorithm

**Figure 2:**
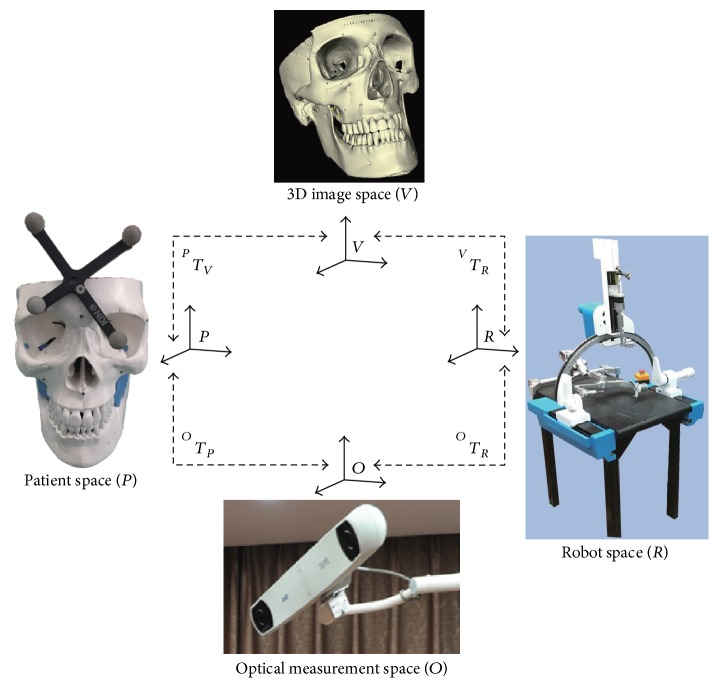
The transformation relationship between coordinate systems.

The spatial registration essentially performed the transformation matrix among the 3D image coordinate system, patient coordinate system, robotic coordinate system, and optical coordinate system [23]. The transformation relationship between coordinate systems is shown in Figure 2.

When the number of point sets is few, a classical ICP algorithm could not get the accurate and stable results, which are needed to iteratively solve the corresponding relation between two point sets. In order to reduce patient suffering caused by placing the titanium screws, the registration process could not provide enough marker points. So the classical ICP algorithm need to be improved to calculate transformation matrixes. In the improved ICP algorithm, the corresponding relation of points between two known point sets should be seen in the initial condition to avoid the iterative solution process between two unknown point sets. Then, solve the mapping relationship by the least squares method and calculate iteratively the transformation matrixes based on the result error, until the accuracy is higher than the certain safety threshold [24–26].

The navigation spatial registration based on the improved ICP algorithm is illustrated as follows [27].

1. ^*O*^*T*_*P*_, which is the transformation matrix from the patient space to optical measurement space, can be obtained by the optical tracker and passive rigid body on the patient skull.
2. ^*P*^*T*_*V*_ is the transformation matrix from the 3D image space to patient space. Firstly, the coordinates of some medical marker points (titanium screws) which were fixed on the patient skull should be obtained by using the optical tracker and passive probe. Through the ^*O*^*T*_*P*_, the above coordinates could be transformed into the patient space. So the coordinates of medical marker points in the patient space could be measured. Based on the coordinates of the medical marker points in the 3D image space and patient space, the transformation matrix ^*P*^*T*_*V*_ could be calculated by the improved ICP algorithm.
3. ^*O*^*T*_*V*_, which is the transformation matrix from the 3D image space to optical measurement space, could be computed from the following formula:

(1) TOV=TOPTPV.

(4) ^*O*^*T*_*R*_, which is the transformation matrix from the robot space to optical measurement space, can be obtained by using the optical tracker and passive rigid body on the robot. The robot coordinate system *R* should be established by the passive rigid body of the robot.

However, the kinematics and control of the robot should be realized in the robot base coordinate system *R*_B_ which is generated in the robot body. So the transformation matrix ^*R*_B_^*T*_*O*_ from the optical measurement space to the robot base coordinate system *R*_B_ should be solved.

First, the local coordinate system *R*_L_ is established through the three points, which are marked on the fixed position of the robot body. Then, the ^*R*_B_^*T*_*R*_L__ could be got, which is the transformation matrix from the robot local coordinate system to the robot base coordinate system. Next, the coordinate of the three points in the optical measurement space is measured by using the passive probe to touch the three points on the surface of the robot, and the transformation matrix ^*O*^*T*_*R*_L__ from the robot local coordinate system to optical measurement space could be calculated. So the mapping relationship ^*R*_B_^*T*_*O*_ between the optical measurement space and robot base coordinate system is measured as follows:

(2) TRBO=TRBRLTORL−1.

Furthermore, the transformation matrix ^*R*^*T*_*R*_B__ from the robot base coordinate system to the robot space could be calculated by the following formula:

(3) TRRB=TOR−1TRBO−1.

As a result, controlling the robot to a certain position could be realized, as long as the corresponding parameters are described in the robot space.

(5) ^*R*^*T*_*P*_, which is the transformation matrix from the patient space to robot space, is calculated by ^*O*^*T*_*R*_ and ^*O*^*T*_*P*_ as follows:

(4) TRP=TOR−1TOP.

(6) ^*V*^*T*_*R*_, which is the transformation matrix from the robot space to 3D image space, is calculated by ^*O*^*T*_*R*_ and ^*O*^*T*_*V*_ as follows:

(5) TVR=TOV−1TOR=TOPTPV−1TOR.

In robot-assisted surgery, the target position and orientation matrix *V*_Target_, which is planned by the surgery planning system in preoperation, can be obtained in the 3D image space. The target matrix *R*_Target_ in the robotic space which corresponds to the target matrix *V*_Target_ can be obtained. According to the above matrix transformation, the equation *R*_Target_ = (^*V*^*T*_*R*_)^−1^*V*_Target_ could be got.

Then, the target matrix *R*_Target_ can be calculated after getting ^*V*^*T*_*R*_, and the robot can move to the target position with right orientation.

#### 2.2.2. Hand-Eye Coordination Based on Optical Positioning

To control the end effector of the robot from the current position to target position, the target position and orientation of the end effector in the robot base coordinate system must be described. After establishing the transformation relationship between the robot base coordinate system and optical measurement space, transform the position and orientation of the end effector into the robot base coordinate system in order to control the end effector to the target position and orientation. This procedure is called the robot hand-eye coordination [28, 29].

**Figure 3:**
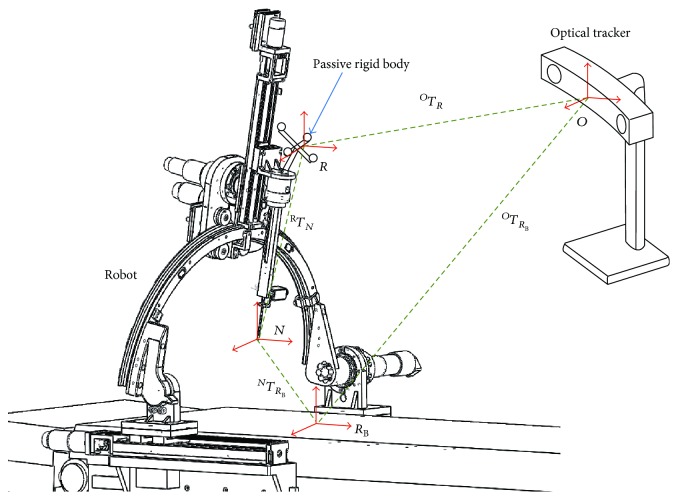
Hand-eye coordination based on optical navigation.

The end effector of the robot is a puncture needle. Because the position of the puncture needle will be changed with the movement of the robot, the passive rigid body is installed at the one end of the end effector. The transformation relationship between the passive rigid body coordinate system and puncture needle coordinate system is fixed. The hand-eye coordination procedure means the relative position and posture between the puncture needle and passive rigid body, which would be calculated. Through the passive rigid body, the optical tracker can get the current and target position and orientation of the puncture needle in the optical measurement space. The different types of puncture needles will be used for different surgeries; we can study the relationship between one puncture needle and the passive rigid body here. The key of hand-eye coordination is to get the ^*R*^*T*_*N*_, which is the transformation matrix from the puncture needle coordination system to robot coordination system. The hand-eye coordination based on optical navigation is shown in Figure 3 and described below.

First, install a needle to the end of the robot. Control the needle tip to touch a point *P* on the calibration block, and we can obtain the corresponding transformation matrix ^*O*^*T*_*R*_. Get the coordinates of *P*_1_(*x*_1_, *y*_1_, *z*_1_) and *P*_2_(*x*_2_, *y*_2_, *z*_2_) on the structure of the end effector by the passive probe, and the direction of $\vec{P_{1}P_{2}}$ is parallel with the needle. Then, remove the puncture needle and obtain the coordinate *P*(*x*, *y*, *z*) in the navigation coordinate system by the passive probe. Establish a needle tip coordinate system with the origin *P*(*x*, *y*, *z*) and the direction $\vec{P_{1}P_{2}}$; then, we can obtain ^*O*^*T*_*N*_, which is the transformation matrix from the puncture needle coordination system to optical coordination system. The transformation matrix ^*R*^*T*_*N*_ can be calculated by ^*O*^*T*_*R*_ and ^*O*^*T*_*N*_ as follows:

(6) TRN=TOR−1TON.

The transformation relationship between the puncture needle coordinate system and robot coordination system can be obtained. And the real-time transformation matrix ^*O*^*T*_*R*_ can be obtained through the optical tracker and passive rigid body. So the relationship between the puncture needle and optical navigation coordinate system can be obtained, and the position and orientation of the puncture needle can be obtained and controlled in real time.

## 3. “Kinematics + Optics” Hybrid Motion Control Method

Before the robot moves to the target point, the target pose of the end effector will be transformed into the parameters of each joint of the robot through inverse kinematics, coordinate system transformation, and medical images. Then, the robot can start moving towards the target point according to the preoperative planning.

In the process of robot moving, the positioning accuracy of the robot could be affected by the kinematics calculation, mechanical transmission, servo control, and so on. And it is difficult to obtain high positioning accuracy by controlling robot motion through kinematics only. In order to improve the positioning accuracy of the robot, we propose the “kinematics + optics” hybrid motion control method. In this method, the robot and its servo controller are considered a servo control system and a control object in the whole system. The navigation system compensates the robot according to the position and orientation deviation of the robot, so as to realize the global closed-loop control. At the same time, the navigation system will track the two passive rigid bodies fixed on the robot and patient's head in real time. Then, the head and the robot can be tracked and compensated in real time to ensure the accuracy of the puncture.

**Figure 4:**
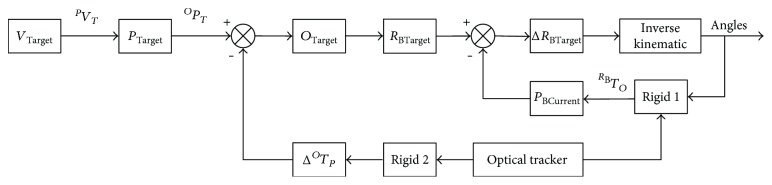
The automatic control system of the robot based on optical navigation.

The principle block diagram of the method is shown in Figure 4. Firstly, the target point in the 3D reconstructed image is converted into the real patient coordinate system. The navigation system is used to locate the position of the patient in real time by the passive rigid body fixed to the patient, so as to compensate the deviation caused by the movement of the patient. After the robot gets the position and orientation of the target, it can drive the robot joints' motion by comparing with the real-time position and orientation of the needle, so as to achieve accurate positioning of the target point.

In the process of robot automatic control, the global closed-loop control is realized by optical navigation. *V*_Target_ is the position and orientation information of the target point in the image coordinate system. *P*_Target_ is the position and orientation information of the target point in the patient coordinate system. *P*_Target_ can be obtained by the position transformation relationship between the patient coordinate system and image coordinate system as follows:

(7) PTarget=TPVVTarget.

The target point is converted from the patient coordinate system to navigation coordinate system, and the conversion relationship between the two coordinate systems has been obtained in the spatial registration above. In actual operation, the patient or the passive rigid body (rigid 2) fixed on the patient may move, which can be compensated by real-time navigation and tracking, and Δ^*O*^*T*_*P*_ is the compensation parameter. *O*_Target_ is the position and orientation information of the target point in the navigation coordinate system. Then, the target point in the navigation coordinate system is as follows:

(8) OTarget=TOP−ΔTOPPTarget.

Before the robot moves to the target point, the target position and orientation of the robot will be transformed from the navigation coordinate system to robot coordinate system. At the same time, the robot or the passive rigid body (rigid 1) fixed on the robot may be moved for some reason, which can be compensated by real-time navigation and tracking, and Δ^*R*_B_^*T*_*O*_ is the compensation parameter. In the robot system, the passive rigid body is installed at the end of the robot, and the conversion relation of the robot is based on the base coordinate system of the robot. *R*_BTarget_ is the target position and orientation information of the needle tip in the robot base coordinate system. Then, the robot base system can be determined by inverse kinematics of the robot. Thus, the deviation between the robot base coordinate system and navigation coordinate system can be calculated. The target point in the robot base coordinate system can be expressed as follows:

(9) RBTarget=TRBO−ΔTRBOOTarget.

Through inverse kinematics calculation, the target point can be converted into the joint vectors of the robot to control the robot motion. The position information of the current puncture point in the navigation coordinate system can be obtained by the real-time tracking of the passive rigid body. The position information of the current puncture point is converted to the robot coordinate system, and the closed-loop control can be achieved by comparing with the target position and orientation information in the robot base coordinate system. Δ*R*_BTarget_ is deviation between the current position and orientation information of the current puncture point and the target position and orientation information in the robot base coordinate system. The deviation can be adjusted by a PID controller:

(10) $$\begin{array}{c} \Delta {\bar{R}}_{BTarget\left(k\right)}=K_{p}\left[E_{\left(k\right)}-E_{\left(k-1\right)}\right]+K_{I}E_{\left(k\right)}+K_{D}\left[E_{\left(k\right)}-2E_{\left(k-1\right)}+E_{\left(k-2\right)}\right],\\ {\bar{R}}_{BTarget\left(k\right)}={\bar{R}}_{BTarget\left(k-1\right)}+\Delta {\bar{R}}_{BTarget\left(k\right)}, \end{array}$$

where *K*_p_ is the proportional gain, *K*_I_ is the integral gain, *K*_D_ is the derivative gain, *k* is the sampling sequence and *k* = 0, 1, 2,…, *n*, and *E*_(*k*)_ is the deviation between the target point and current position. $\Delta {\bar{R}}_{BTarget\left(k\right)}$ is the increment of deviation at the *k* sampling time. ${\bar{R}}_{BTarget\left(k\right)}$ is the output of the PID controller at the *k* sampling time. The optimal deviation is obtained by adjusting the gain parameters of the PID controller [30]. Thus, the joint angles can be calculated by inverse kinematics to control the robot to reach the target position.

## 4. Experiments and Results

### 4.1. System Model Tests

#### 4.1.1. Optical Measuring Instrument Accuracy Test

Because the navigation system is based on optical measurement, the accuracy of the optical measuring instrument has a great influence on the performance of the navigation system. Therefore, the positioning accuracy of the optical measuring instrument has been tested as follows.

**Figure 5:**
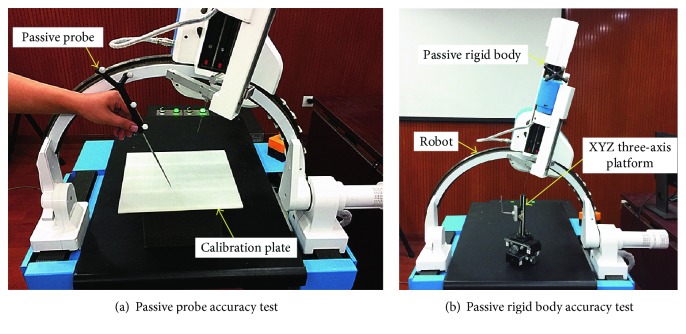
Optical measuring instrument accuracy test.

First, the positioning accuracy of the passive probe is tested, as shown in Figure 5(a). The calibration plate is used as a position-measuring device. There is a horizontal and vertical scale of 5 mm absolute spacing on the calibration plate. In the test, place the calibration plate in the measured position in any direction and angle. Any two points on the calibration plate were selected as the test points of the passive probe. The two points are touched, respectively, by the passive probe perpendicular to the calibration plate, and the coordinates of the two points under the optical measuring device are obtained. The distance between the two points in the actual and optical measuring device is computed, respectively, and the difference of distance is the positioning error of the passive probe. Repeat the above procedure 20 times.

Then, the positioning accuracy of the passive rigid body is tested, as shown in Figure 5(b). In the test, the XYZ three-axis platform is used as a position-measuring device. The measurement accuracy of the XYZ three-axis platform is 0.1 mm. The passive rigid body is fixed at the end effector of the robot. The end effector is controlled to move a series of fixed distances, and its coordinates are recorded by the XYZ three-axis platform simultaneously. Repeat the above procedure 20 times.

In this section, the accuracy of the optical measuring instrument is verified by experiments. Experimental results show that the average positioning error of the passive probe is 0.09 mm. The average positioning error of the passive rigid body is 0.2 mm. The experimental errors of the optical measuring instrument are within the nominal error range of 0.25 mm, which can meet the needs of the surgical robot system.

#### 4.1.2. Registration Accuracy Test

The surgical planning path can be converted to the robot puncture path through the registration between the 3D reconstructed image and patient. The registration accuracy has a great influence on the accuracy of the robot system, so it is necessary to verify the accuracy of the registration algorithm through experiment. The lesion area of puncture operation is usually in the skull base, but the registration marker points can be placed on the surface of the skull usually. Therefore, the registration can be achieved through eight marker points outside the skull, and the registration error can be verified by the other ten marker points. The eight registration marker points are located in the middle of eyebrows, anterior nasal spine, left orbit, right orbit, left infraorbital, right infraorbital, left external auditory meatus, and right external auditory meatus. The ten registration verification marker points are located in the both sides of the subtemporal, foramen ovale, external carotid artery, jugular foramen, and styloid process.

**Figure 6:**
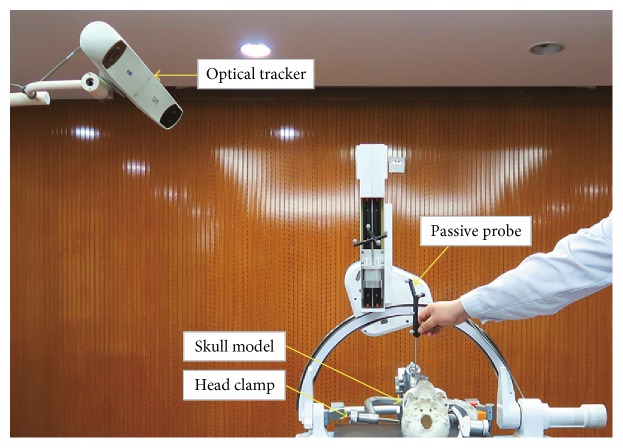
The registration accuracy test.

As shown in Figure 6, the optical tracking system, which contains the optical tracker and passive probe, is used as a measurement tool in this experiment. The skull model marked with medical titanium screws (1.5 mm × 5 mm) is the experimental object. The head clamp is used for fixing the skull model. The Cone beam CT (CBCT) NewTom VG is used to obtain the patient's medical image. The graphic workstation is used for performing the 3D image reconstruction. The work flow of the registration accuracy experiment is shown as follows.

1. The medical titanium screws were drilled into the eight registration marker points and ten registration verification marker points of the skull model.
2. The medical image of the skull model was obtained by CBCT, and the image was imported into the graphic workstation for 3D image reconstruction.
3. Fix the skull model on the experimental platform with the head clamp, and put the navigation equipment in the right position.
4. The spatial coordinates of eight registration points were acquired by the passive probe.
5. In the 3D medical image, the coordinates of corresponding registration points are acquired in order. The conversion relationship between the 3D image coordinate space and the optical navigation coordinate space is obtained by performing the registration. And the conversion relationship can be represented by *T*.
6. The spatial coordinates of ten verification points on the skull model are obtained by using the passive probe, and the coordinates were recorded as *A*(*A*_*x*_, *A*_*y*_, *A*_*z*_). In the 3D medical image, the coordinates of corresponding verification points are acquired in order and the coordinates were recorded as *B*(*B*_*x*_, *B*_*y*_, *B*_*z*_). The coordinates of points on the medical image are converted to the optical navigation coordinate system, and the coordinates can be recorded as follows:

(11) $$\begin{array}{c} C\left(C_{x},C_{y},C_{z}\right)=TB\left(B_{x},B_{y},B_{z}\right). \end{array}$$

(7) The Euclidean distance between the corresponding points *A* and *C* is analyzed and compared, and the Euclidean distance was recorded as |*AC*|. |*AC*| is the registration error of the verification point and can be calculated as follows:

(12) $$\begin{array}{c} \left|AC\right|=\sqrt{{\left(A_{x}-C_{x}\right)}^{2}+{\left(A_{y}-C_{y}\right)}^{2}+{\left(A_{z}-C_{z}\right)}^{2}}. \end{array}$$

(8) Repeat the registration process six times, and record and analyze the registration error.

The experimental results are shown in Table 1. Mean represents the average error, and SD represents the standard deviation of errors. According to the experimental data, the average of the registration error of each target point in each group of experiments and the average of the registration error of the same target point in many groups of experiments are obtained, and they are all less than 0.75 mm. The corresponding standard deviations are small also. It can be seen that the registration algorithm can be applied to image registration between the 3D medical image and patient accurately and stably.

### 4.2. Positioning Accuracy Tests

In this section, two positioning accuracy tests have been implemented: (1) the positioning error of the open-loop robotic control and (2) the positioning error of the closed-loop robotic control. Test 1 can verify the work ability of the robot. By comparing tests 1 and 2, the feasibility of the proposed “kinematics + optics” hybrid motion control method can be proved.

**Figure 7:**
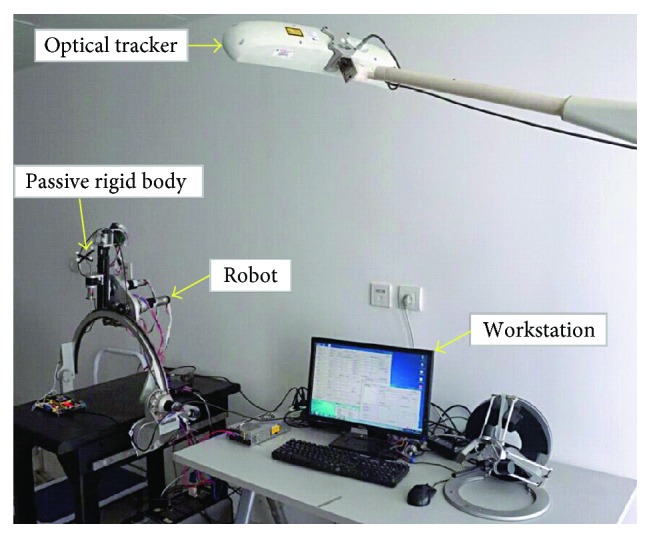
Measurement of positioning accuracy.

In the first positioning accuracy test, the optical measuring instrument is used as a position-measuring device, as shown in Figure 7. Before testing, the passive rigid body is fixed at one end of the end effector and the optical tracker is placed in the corresponding position. The robot is controlled by the servo motion control system. The end effector is controlled to move a series of predetermined target positions, and its coordinates are recorded by the optical measuring instrument simultaneously. The positioning error of the robot can be calculated by the deviation between the given target point and the actual point of arrival of the end effector.

In the second positioning accuracy test, the test platform is the same as Section 4.1.1, as shown in Figure 5(b). In the test, the passive rigid body is fixed at the end effector of the robot. The optical tracker is placed in a representative location to achieve the best positioning effect. The “kinematics + optics” hybrid motion control method is applied to the motion control of the robot. The end effector of the robot is controlled to move a series of predetermined target positions, and its coordinates are recorded by the XYZ three-axis platform simultaneously. Then, the positioning error of the robot based on the “kinematics + optics” hybrid motion control method can be obtained.

**Figure 8:**
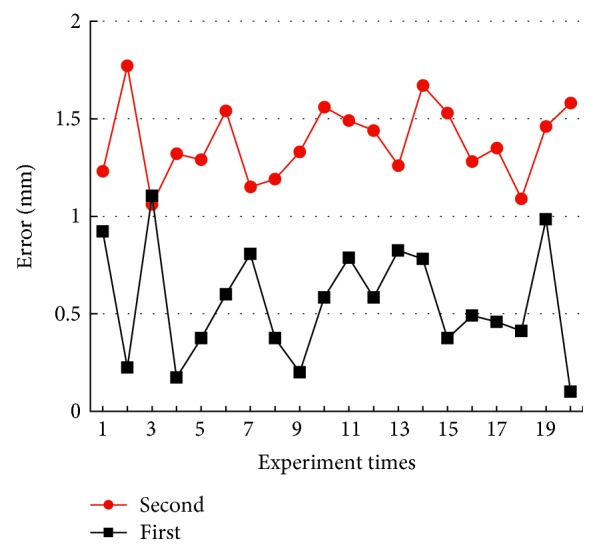
The results of the positioning accuracy test.

Repeat the above tests 20 times, respectively. The results of two positioning accuracy tests are shown in Figure 8. In test 1, the average positioning error *E*_1_ is 1.38 mm and the maximum error is 1.77 mm. In test 2, the average positioning error *E*_2_ is 0.56 mm and the maximum error is 1.10 mm. The result *E*_1_ > *E*_2_ shows that the motion control method presented in this paper can improve the average positioning accuracy at least 55%. Meanwhile, compared with test 1, the biggest experimental error is reduced from 1.77 to 1.10 mm, as shown in Figure 8. In general, 1.10 mm is smaller than the diameter of the lesion, so the risk of positioning deviation can be eliminated and the method is feasible.

### 4.3. Skull Model Experiment

The robot system based on optical navigation can assist surgeons to complete three kinds of surgeries, including biopsy of skull base lesions, radiofrequency thermocoagulation of the trigeminal ganglion, and radioactive particle implantation of skull base malignant tumors. In order to verify the universality and feasibility of the system model and control method based on optical navigation in the whole robot system, the skull model experiments are carried out on the three kinds of surgeries. And the skull model experiments are performed by the surgeon himself.

#### 4.3.1. Model Experiment of Biopsy of the Skull Base Lesions

Biopsy is a common procedure in the medical field, which can effectively diagnose the lesion area. There are two major approaches for biopsy, needle biopsy and open excisional biopsy, and the needle biopsy is more attractive in surgery [31]. While in most cases the needle biopsy can be completed without difficulty, there are limitations to the accuracy obtainable using freehand techniques [32]. In order to improve the effect of needle biopsy, we use the robot system to assist surgeons to complete the needle biopsy, and the related experiment is as follows [33].

**Figure 9:**
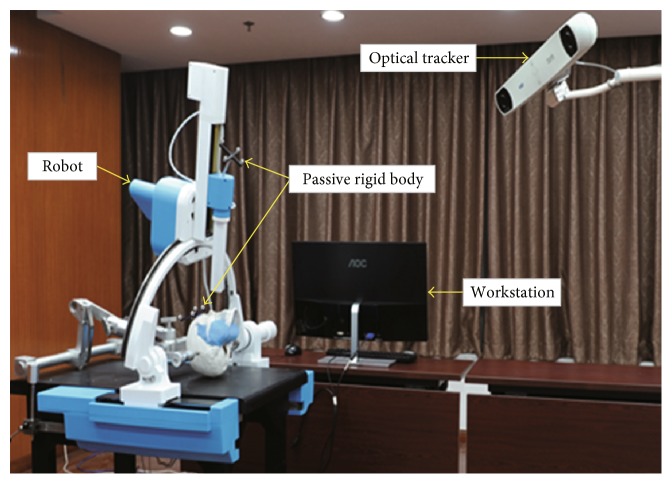
The skull model experimental platform.

The devices and materials used in the experiment include the surgical robot, a skull model, the meatball, the Cone beam CT, the super light clay, the biopsy gun, the biopsy needle, and the medical titanium screws. And in the skull model experiment, the meatball is used as the lesion tissues. The experimental platform is shown in Figure 9. The work flow of the experiment is shown as follows.

1. The meatball with super light clay was fixed in the skull model as the lesion tissue. The titanium screws were fixed on the surface of the skull model as the registration marker points.
2. The medical image of the skull model was obtained by CBCT, and the image was imported into the graphic workstation for 3D image reconstruction.
3. Get the position of the meatball, and complete the surgical planning of biopsy in the 3D medical image.
4. Based on the optical tracker and passive probe, the registration between the robot body and the optical navigation system is completed. The end effector of the biopsy is mounted on the robot.
5. The skull model is fixed on the operation platform with the head clamp. The corresponding coordinates of the titanium screw marker points in the optical navigation coordinate system and the medical image coordinate system are obtained. Medical software is used to complete the registration between the medical image and optical navigation system.
6. According to the surgical planning, the coordinates of the puncture target and insertion point outside the skin were transferred to the robot coordinate system. The end of the robot is moved to the vicinity of the insertion point, and the automatic control model is used to realize the accurate positioning of the needle.
7. The robot performs the puncture operation automatically. When the robot reaches the target position, the system indicates that the puncture is over.
8. Press the button of the biopsy gun to complete biopsy. Remove the puncture biopsy needle from the end effector, and maintain the biopsy needle at the location where the puncture is completed. Control the end of the robot to return to its initial position.
9. Scan the skull model with the puncture needle, and import the data into the medical software to reconstruct the 3D model again. The reconstructed 3D model was fused with the preoperative medical image to calculate the distance between the target point and the needle tip, and record the results.

**Figure 10:**
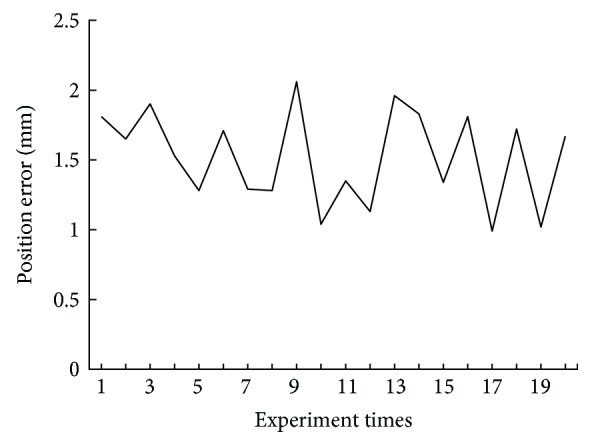
The results of 20 groups of biopsy experiment.

**Figure 11:**
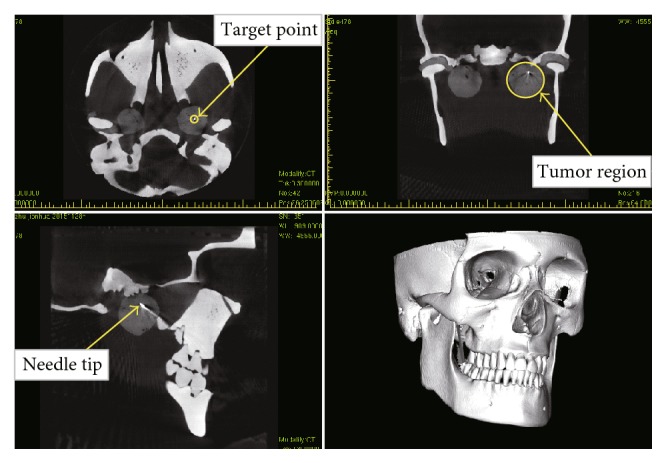
Postoperative CT scan results.

According to the above procedure, 20 groups of biopsy experiment were performed the results are shown in Figure 10. The average puncture error of the system is 1.52 mm, and the standard deviation is 0.33 mm. In each group, the target tissue can be successfully obtained and the postoperative CT scan results are shown in Figure 11.

#### 4.3.2. Model Experiment of Radiofrequency Thermocoagulation of the Trigeminal Ganglion

Radiofrequency thermocoagulation of the trigeminal ganglion is an effective method for the treatment of trigeminal neuralgia and is one of the common operations in cranio-maxillofacial surgery. The operation uses a puncture needle to puncture the position of the trigeminal ganglion and carries on radiofrequency thermocoagulation therapy to the target. The trigeminal ganglion is located inside the foramen ovale. During the puncture, the puncture needle must pass through a foramen ovale with a diameter of about 5 mm to reach the trigeminal ganglion [34]. Therefore, surgeons usually use the needle through the foramen ovale as a sign of successful operation.

**Figure 12:**
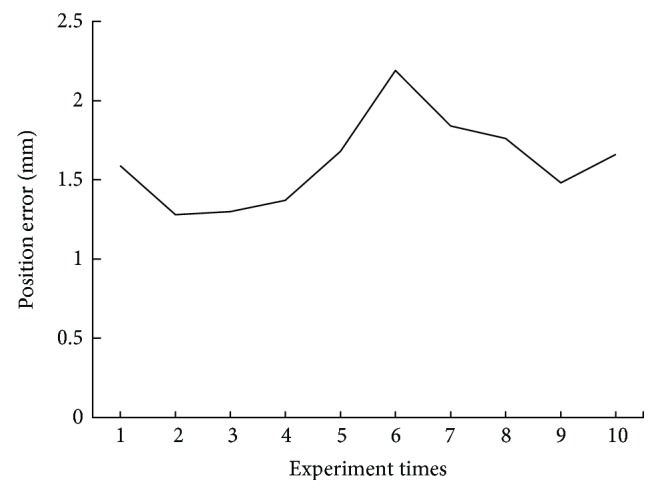
The puncture error of model experiment.

The operation procedure of the trigeminal thermocoagulation experiment is exactly the same as that of the biopsy experiment, but the end effector is different. The procedure of operation of the trigeminal thermocoagulation can be referred to the biopsy. In the model experiment, 10 groups of trigeminal thermocoagulation experiment were performed; the experimental results are shown in Figure 12. The average puncture error of the model experiment is 1.62 mm, the standard deviation is 0.26 mm, and the puncture needle can pass through the oval hole every time. The experimental results show that the success rate of the robotic system in the model experiment of radiofrequency thermocoagulation of the trigeminal ganglion is 100%.

#### 4.3.3. Model Experiment of Radioactive Particle Implantation

Radioactive particle implantation experiment of skull base malignant tumor was also performed according to the actual operation [35]. First, the tumor is segmented from the medical image, and then, the particle implantation path is automatically generated on the tumor. Surgeons can adjust the particle implantation path according to experience. The robot can carry out the implantation of radioactive particles according to path planning. In the model experiment, the meatball is also uses as the tumor tissues. The radioactive particles are replaced by the steel wires of 5 mm in length, and the size of steel wires are the same as the size of radioactive particles. The particles were implanted with an automatic particle gun, and the rationality of particle implantation was verified. In addition to the basic position error, the TPS system is used to verify the rationality of particle implantation through the deviation of the radioactive dose topographic map. Due to the limited size of the meatball, three paths were designed in the model experiment and each path was implanted with 5 particles.

The devices and materials used in the experiment include the surgical robot system, the automatic particle implantation device, the steel particles, the meatball, the CBCT, the super light clay, the titanium screws, and the skull model. The work flow of the experiment is shown as follows.

**Figure 13:**
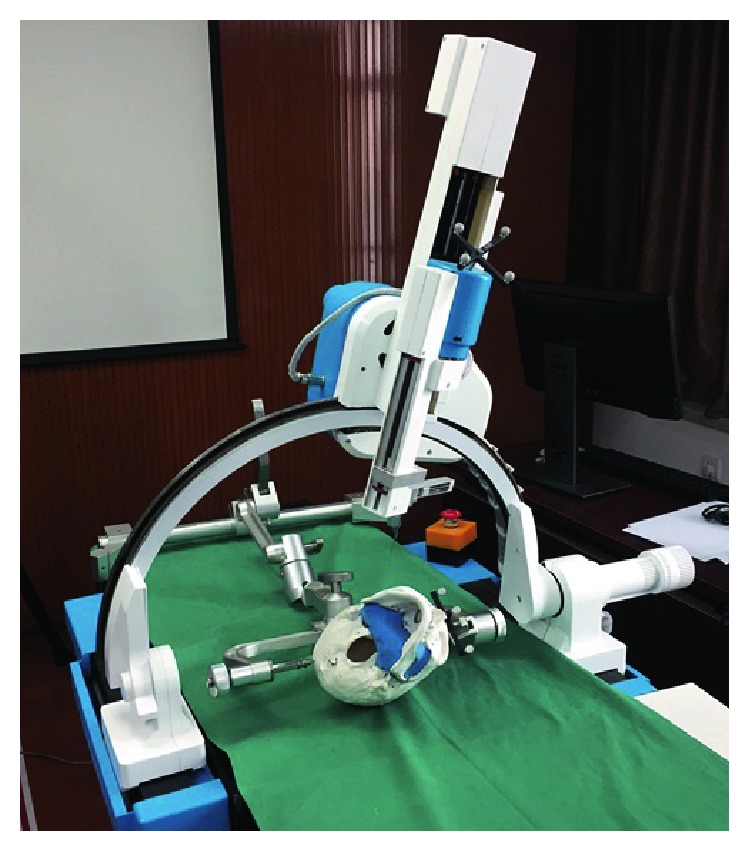
The radioactive particle implantation experiment.

1. Load the particles into the automatic particle implantation gun.
2. The meatball with super light clay was fixed in the skull model as the tumor tissues. The titanium screws were fixed on the surface of the skull model as the registration marker points.
3. The 3D reconstructed image of the skull model was obtained by CBCT and the graphic workstation. Find out the tumor from the 3D reconstructed image, and automatically generate the particle implantation path. Surgeons can adjust the particle implantation path according to experience.
4. The optical navigation system is used to complete the spatial registration of the 3D reconstructed image space, the robot space, the optical navigation space, and the patient space.
5. The robot was automatically controlled to reach the puncture point of the first path and complete the puncture. According to the number and spacing of particle implantation on the first path, the robot system implants particles sequentially. After the robot completes the particle implantation, it automatically returns to the insertion point, as shown in Figure 13.
6. Similarly, refer to the above steps to complete the implantation of particles in the other two paths.
7. After completion of the particle implantation, the skull model was scanned by CBCT and analyzed.

**Figure 14:**
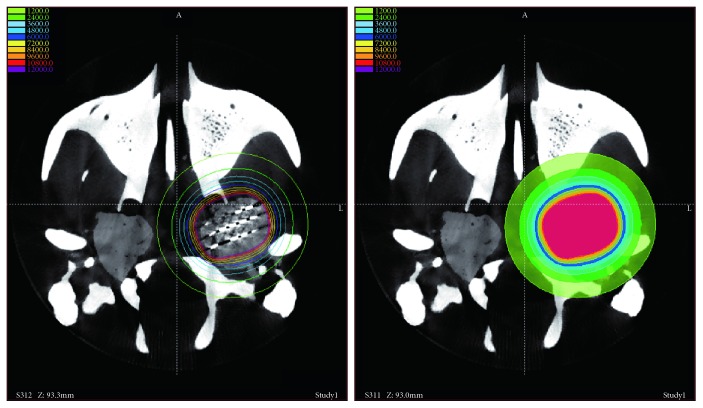
The radioactive dose topographic map.

In the model experiment, 10 groups of particle implantation were performed. The average error of particle implantation is 1.51 mm, and the standard deviation is 0.50 mm. The effect of particle implantation is usually analyzed by the TPS system. Assuming that the implanted particles are radioactive particles, the corresponding verification results of the TPS system are shown in Figure 14. The results of TPS system analysis show that the radiation dose map deviation is less than 1%, which means that particle implantation is reasonable.

## 5. Conclusions and Discussions

This paper presents a novel cranio-maxillofacial puncture robot system through the analysis of surgical requirements, and the system can assist surgeons to complete biopsy of skull base lesions, radiofrequency thermocoagulation of the trigeminal ganglion, and radioactive particle implantation of the maxillofacial tumor. The surgical robot system is divided into four parts, including the 3D reconstructed image subsystem, robotic subsystem, optical measurement subsystem, and patient subsystem. The spatial registration based on quaternion and the iterative closest point registration algorithm is realized to obtain the transformation relationship between four subsystems. The hand-eye coordination model is established to control the end effector of the robot moving to the target position according to preoperative planning. The “kinematics + optics” hybrid motion control method is presented to improve the positioning accuracy of the robot end effector. The accuracy of the system model was measured through the model tests. And the feasibility of the “kinematics + optics” hybrid motion control method was verified by comparing the positioning accuracy before and after the application of the method. Finally, the skull model experiments were carried out to evaluate the function of the whole robot system. The experimental results show that the feasibility and effectiveness of the robot system based on optical navigation can meet the needs of cranio-maxillofacial surgery.

During the puncture process, imaging data can provide precise navigation for precise needle placement. Along with CT fluoroscopy [17–19], intraoperative ultrasound [20], and MRI [21], optical navigation is also now an important tracking system for real-time guidance. Real-time vision feedback can verify the needle's advancement along the planned path without intraoperative X-ray exposure to the patient. Accuracy depends on the uninterrupted update of position data acquired by optical tracking, which provides accurate vision feedback and closed-loop control. And the navigation system can track the two passive rigid bodies fixed on the robot and patient's head, respectively, in real time. Then, the robot and head can be tracked and compensated in real time to ensure the accuracy of puncture. However, infrared signals can only be transmitted in straight lines, so the optical measuring instrument should have good visibility when used.

The accuracy requirement for cranio-maxillofacial puncture is determined by the characteristics of lesions in different surgeries. However, there is no generally agreed value currently. According to the surgeon's clinical experience and related literature [36, 37], select 3 mm as the accuracy limit. And this is also the minimum safety distance to protect the carotid artery for this robot system in the preoperative planning. In each of the above three surgical skull model experiments, the average puncture error of the robot system is less than 3 mm. Using the proposed navigation system and the control method in this paper, we consider our result to be meeting the accuracy requirement. The results of three skull model experiments are similar, indicating that the system has good consistency.

Although the results of this paper has showed the feasibility of the robot system based on the navigation system, there are still a number of shortcomings and designs needed to be overcome, improved, and optimized. The method of fixing titanium screws on the patient for registration is invasive, and the noninvasive methods should be proposed. In the optical tracking system, the end effectors of the robot cannot be displayed in the surgical design system in real time, which is adverse to carry out the real-time navigation of the robot for surgeons. More experiments will be done in animal models and humans to detect and evaluate the feasibility of the robot system. The safety of the robot system should be improved and optimized. More flexible robot wrist and more easy-to-use end effectors need to be designed according to clinical requirements.

## Figures and Tables

**Table 1:** The results of the registration accuracy test.

The target point	The registration error (mm)						
	Group one	Group two	Group three	Group four	Group five	Group six	Mean ± SD
Right subtemporal	0.70	0.67	0.63	0.66	0.65	0.45	0.63 ± 0.08
Right foramen ovale	0.66	0.78	0.84	0.73	0.75	0.52	0.71 ± 0.10
External orifice of the right carotid artery	0.42	0.62	0.50	0.55	0.63	0.72	0.57 ± 0.10
Right jugular foramen	0.54	0.76	0.82	0.68	0.69	0.62	0.69 ± 0.09
Right styloid process	0.35	0.93	0.41	0.72	0.77	0.55	0.62 ± 0.20
Left subtemporal	0.51	0.86	0.73	0.35	0.59	0.77	0.64 ± 0.17
Left foramen ovale	0.68	0.78	0.69	0.71	0.87	0.68	0.74 ± 0.07
External orifice of the left carotid artery	0.72	0.79	0.71	0.62	0.65	0.38	0.65 ± 0.13
Left jugular foramen	0.54	0.66	0.95	0.45	0.92	0.81	0.72 ± 0.19
Left styloid process	0.72	0.57	0.63	0.54	0.59	0.92	0.66 ± 0.13
Mean ± SD	0.58 ± 0.13	0.74 ± 0.11	0.69 ± 0.15	0.60 ± 0.12	0.71 ± 0.11	0.64 ± 0.16	—
